# (-)-α-Bisabolol Alleviates Atopic Dermatitis by Inhibiting MAPK and NF-κB Signaling in Mast Cell

**DOI:** 10.3390/molecules27133985

**Published:** 2022-06-21

**Authors:** Guangxia Li, Huayan Wu, Liqin Sun, Kang Cheng, Zhi Lv, Kaixian Chen, Fei Qian, Yiming Li

**Affiliations:** 1 School of Pharmacy, Shanghai University of Traditional Chinese Medicine, 1200 Cailun Road, Shanghai 201203, China; lgx1344892@163.com (G.L.); 18283017273@163.com (H.W.); aman1607@163.com (L.S.); kxchen@simm.ac.cn (K.C.); 2 Institute of Interdisciplinary Integrative Medicine Research, Shanghai University of Traditional Chinese Medicine, 1200 Cailun Road, Shanghai 201203, China; 3 Shanghai Inoherb Cosmetics Co., Ltd., Shanghai 200080, China; chengkang@inoherb.com (K.C.); luzhi@inoherb.com (Z.L.)

**Keywords:** atopic dermatitis, mast cells, JNK, NF-κB, (-)-α-Bisabolol

## Abstract

(-)-α-Bisabolol (BIS) is a sesquiterpene alcohol derived mostly from *Matricaria recutita* L., which is a traditional herb and exhibits multiple biologic activities. BIS has been reported for treatment of skin disorders, but the effect of BIS on anti-atopic dermatitis (AD) remains unclear. Therefore, we investigated the effects of BIS on 2,4-dinitrochlorobenzene (DNCB)-induced AD in BALB/c mice and the underlying mechanism in Bone Marrow-Derived Mast Cells (BMMCs). Topical BIS treatment reduced AD-like symptoms and the release of interleukin (IL)-4 without immunoglobulin (Ig)-E production in DNCB-induced BALB/c mice. Histopathological examination revealed that BIS reduced epidermal thickness and inhibited mast cells in the AD-like lesions skin. Oral administration of BIS effectively and dose-dependently suppressed mast-cell-mediated passive cutaneous anaphylaxis. In IgE-mediated BMMCs, the levels of β-hexosaminidase (β-hex), histamine, and tumor necrosis factor (TNF)-α were reduced by blocking the activation of nuclear factor-қB (NF-қB) and c-Jun N-terminal kinase (JNK) without P38 mitogen activated protein (P38) and extracellular regulated protein kinases (Erk1/2). Taken together, our experimental results indicated BIS suppresses AD by inhibiting the activation of JNK and NF-κB in mast cells. BIS may be a promising therapeutic agent for atopic dermatitis and other mast-cell-related diseases.

## 1. Introduction

Atopic dermatitis (AD) is a recurrent allergic skin disease caused by some environmental factors and genetic background [1]. AD patients suffer from skin barrier dysfunctions, including severe edema, pruritus, lichenification, dryness, erythema, and excoriation [2], which imposes a significantly reduced quality of life [3,4] along with psychological distress as well as placing substantial burdens on patients and their families. At present, the first-line treatment option for patients with AD is still steroid anti-inflammatory drugs, such as corticosteroids (e.g., prednisolone, dexamethasone, and fludrocortisone). Although these corticosteroids alleviate AD symptoms, they are associated with serious adverse effects, for instance, red burning skin, thinning skin, atrophy, and growth retardation [5,6]. Therefore, increasing attention has been focused on identifying and developing safe and effective drugs for the treatment of AD, especially from plant extracts.

The pathogenesis of AD remains incompletely elucidated. Studies have shown that infiltration of immune cells (e.g., dendritic cell subtypes, T cells, and mast cells) is increased in AD lesions [7,8]. These immune cells are involved in AD as follows: in the context of altered epidermal barrier, antigens are taken up by epidermal Langerhans cells and then Langerhans cells present antigens to T-helper (Th) 0 cells, resulting cytokine secretion. With the release of interleukin (IL) 4, naive T-cells develop into Th2 cells, producing IL-4, IL-5, and tumor necrosis factor (TNF)-α. These Th2 cytokines further activate B cells so as to promote immunoglobulin (Ig)E production. Finally, mast cells are activated by binding to IgE [9,10,11]. Notably, mast cells are effectors of allergic diseases, which are critical for the development of AD [12,13]. Mast cells are activated by multiple stimuli, such as antigens, C3a, C5a, substance P, and LPS [14], and then release various mediators, including preformed granule-associated chemical mediators, lipid mediators, and *de novo* synthesized cytokines. Through these mediators, the inflammatory response in skin is continuously enhanced and persists [15,16]. Therefore, decreasing the activity of mast cells may improve skin symptoms of AD.

Chamomile (*Matricaria recutita* L.) is known as Uygur medicine chamomile, which has been included in Drug Quality Standard of Ministry of Health of the People’s Republic of China (Uygur Medicine). The whole plant is used as medicine, and it exhibits anti-inflammatory, analgesic, and antibacterial properties [17]. It is widely used for skin pruritus and edema in clinics. In addition, chamomile, when associated to a vehicle with emollient function, improved atopic dermatitis (AD)-like lesions in a murine model [18].

(-)-α-Bisabolol (BIS), a sesquiterpene alcohol mostly present in essential oils of chamomile [19], has a variety of biological activities, including antioxidant, gastroprotective, anti-infection, and anti-cancer properties [20,21,22,23,24], among which it has been found to have high anti-inflammatory potential in various disease models. For example, BIS suppresses murine osteoarthritis and advanced glycation end product (AGE)-induced inflammatory reaction in chondrocytes [25]. BIS also can alleviate myocardial infarction by attenuating inflammation via inhibiting NLPR3 inflammasome activation [19]. In addition, a study has shown that atopiclair (Zarzenda), which contains BIS, is an anti-inflammatory agent for the treatment of allergic diseases of the skin [26]. However, the role of BIS in mast cells in AD-like skin lesions remains unclear. Therefore, we investigated the role of BIS in AD both in vitro and in vivo and sought to understand the mechanisms through which BIS regulates allergic inflammation. Our findings indicate that BIS improved AD symptoms by inhibiting mast-cell activation via mitogen-activated protein kinase (MAPK) and nuclear factor-қB (NF-κB) pathways.

## 2. Results

### 2.1. BIS Ameliorated AD-like Skin Symptoms

**Figure 1:**
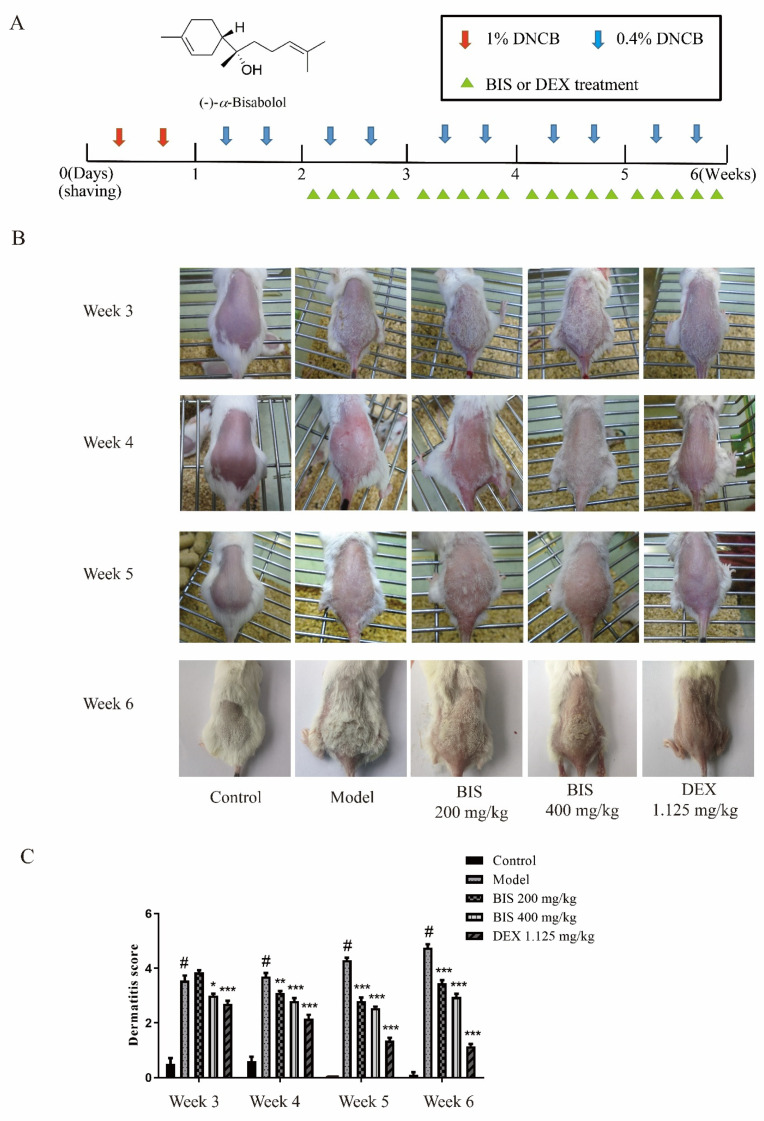
Chemical structure of BIS and effect of BIS on DNCB-induced AD-like skin symptoms. (**A**) Chemical structure of BIS and schematic diagram of the experimental schedule. (**B**) Clinical characteristics of each group on week 3–6. (**C**) The dermatitis scores of the AD-like skin lesions according to four major classes of symptoms. Data represent the mean ± SEM, *n* = 10 mice/group. ^#^ *p* < 0.05 vs. Control; * *p* < 0.05, ** *p* < 0.01, *** *p* < 0.001 vs. Model.

**Figure 2:**
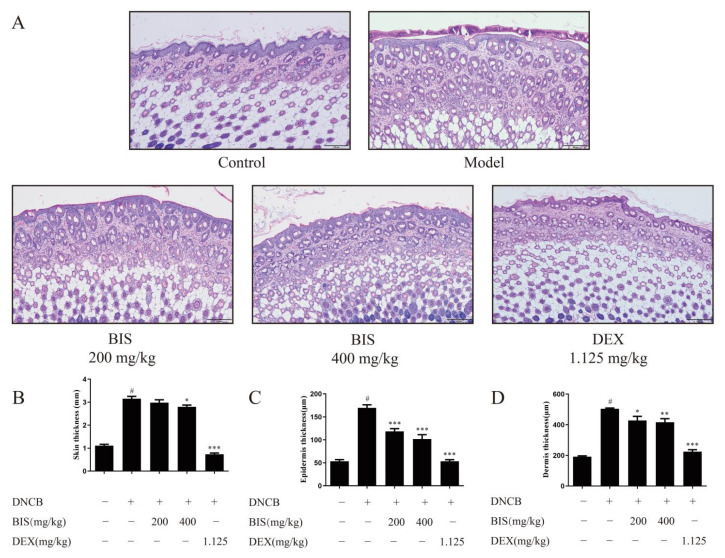
Effects of BIS on DNCB-induced skin thickness. (**A**) The representative images of dorsal skin stained with H&E. (**B**) Measurement of skin thickness by Vernier caliper. Epidermal thickness (**C**) and dermal thickness (**D**) were determined by H&E staining. Magnification: ×100. Scale bar, 200 µm. Data represent the mean ± SEM, *n* ≥ 5 mice/group. ^#^ *p* < 0.05 vs. Control; * *p* < 0.05, ** *p* < 0.01, *** *p* < 0.001 vs. Model.

First, we measured the effect of BIS on AD by DNCB-induced AD mice with the experimental schedule in Figure 1A. After stimulation with DNCB, the dorsal skin exhibited erythema/hemorrhage, edema, and scaling/dryness and was constantly aggravated during the experiment, whereas the dorsal skin, which was treated with BIS, was ameliorated (Figure 1B). Then, we used the dermatitis score to analyze the severity of AD-like skin lesions. As shown as Figure 1C, topical administration of BIS significantly reduced the dermatitis score compared with DNCB-sensitized BALB/c mice. Repeated stimulation with DNCB caused potent inflammatory changes, such as redness, hemorrhaging, and thickening of the dermis and epidermis in the AD mice. Here, the dorsal skin sections were stained and observed under an optical microscope (Figure 2A). Administration of DNCB significantly increased the skin thickness (Figure 2B) compared with normal mice. However, the thickness of both the epidermis (Figure 2C) and dermis (Figure 2D) were improved after treatment with BIS; in particular, BIS significantly reduced the thickness of the epidermis in a concentration-dependent manner.

### 2.2. BIS Relieved Abnormal Immune Response

**Figure 3:**
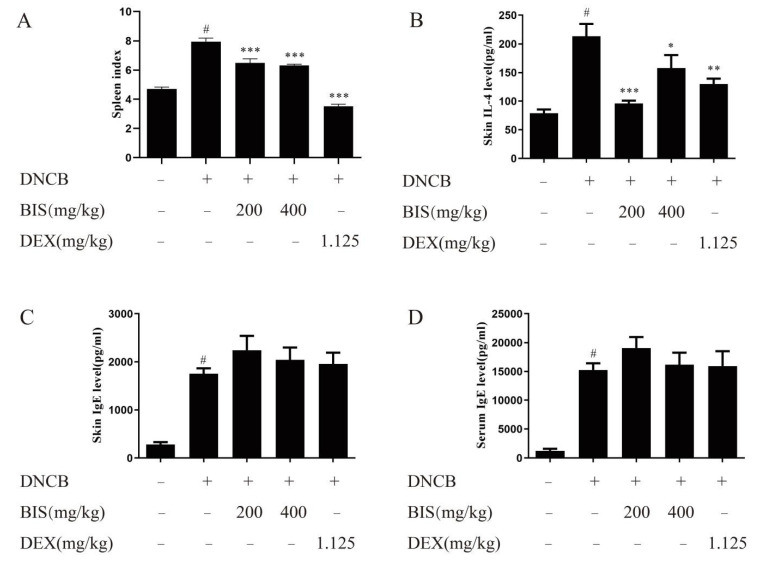
BIS relieved abnormal immune response in AD-like mice. (**A**) Measurement of spleen index by calculating spleen weight/body weight. Skin levels of IL-4 (**B**), skin levels of IgE (**C**) and serum levels of IgE (**D**) were measured by ELISA. Data represent the mean ± SEM, *n* ≥ 8 mice/group. ^#^ *p* < 0.05 vs. Control; * *p* < 0.05, ** *p* < 0.01, *** *p* < 0.001 vs. Model.

The spleen index is an important indicator for reflecting the abnormal immune activation in the AD mouse model [27]. As shown as Figure 3A, BIS dose dependently reduced the spleen index, which was calculated by weight of spleen/weight of body × 100%. Then, we found that BIS attenuated the skin IL-4 release (Figure 3B), which is a Th2 cytokine, and increased in patients with dermatitis [28]. Additionally, we examined the effect of BIS on the release of IgE. Interestingly, the results showed that BIS did not reduce IgE production neither in the dorsal skin (Figure 3C) nor serum (Figure 3D).

### 2.3. BIS Inhibited Mast-Cell Infiltration and Degranulation

**Figure 4:**
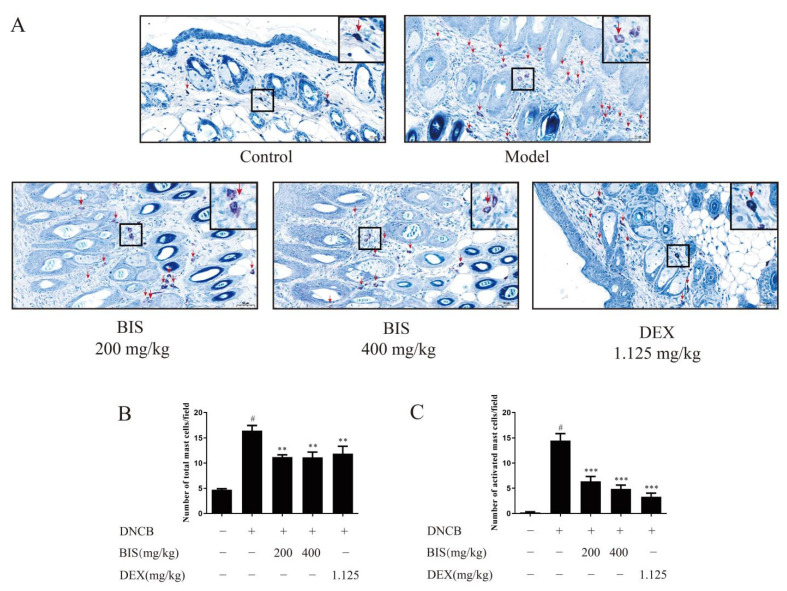
Effect of BIS on mast-cell infiltration and degranulation. DNCB-induced dorsal skin was detected by toluidine blue staining. (**A**) Representative images of skin sections. Red arrow indicated individual mast cell. The number of total mast cells (**B**) and activated mast cells (**C**) were counted of skin sections. Magnification: ×200. Scale bar = 50 µm. Data represent the mean ± SEM, *n* = 5 mice/group. ^#^ *p* < 0.05 vs. Control; ** *p* < 0.01, *** *p* < 0.001 vs. Model.

Given that BIS modulated the AD-like skin symptoms without inhibiting IgE production, we speculate that BIS may alleviate AD symptoms by inhibiting mast-cell activation. Therefore, we used toluidine blue staining in the dorsal skin to observe mast-cell features. Compared with the DNCB-sensitized group, BIS reduced the infiltration of mast cells and the number of mast cells in the dorsal skin of BALB/c mice (Figure 4A,B). Furthermore, topical administration of BIS suppressed mast-cell activation in a concentration-dependent manner (Figure 4A,C).

### 2.4. BIS Suppresses IgE-Induced Anaphylactic Responses in ICR Mice

**Figure 5:**
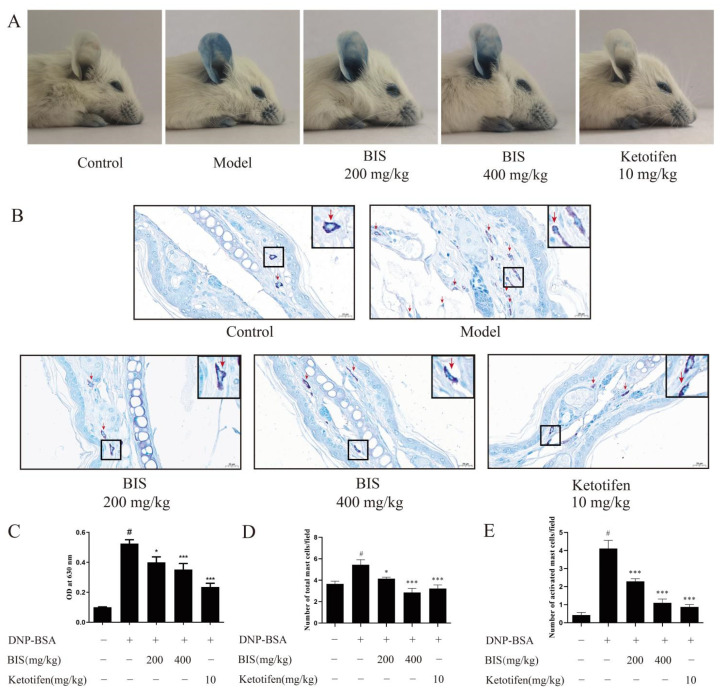
Effect of BIS on IgE-mediated PCA reaction. Mice were sensitized by intradermal injection of anti-mouse-IgE in the ear and stimulated by intravenous injection of DNP-BSA, during which BIS or ketotifen was orally administered. (**A**) Representative photos of ears showing dye extravasation. (**B**) The number of mast cells in the ear was detected by toluidine blue staining. Red arrows indicate individual mast cells. (**C**) Evans blue was extracted with 300 µL of formamide and measured absorbance at 630 nm. The number of total mast cells (**D**) and activated mast cells (**E**) were counted in ear sections. Magnification: ×200. Scale bar = 50 µm. Data represent the mean ± SEM, *n* ≥ 7 mice/group. ^#^ *p* < 0.05 vs. Control; * *p* < 0.05, *** *p* < 0.001 vs. Model.

To further confirm the effect of BIS in improving AD via mast cells, we used Passive Cutaneous Anaphylaxis (PCA) in the ear to evaluate the action of BIS in anaphylactic responses. Mice were sensitized by intradermal injection of anti-DNP-IgE into the ear and stimulated by intravenous injection of DNP-BSA, during which BIS or ketotifen was orally administered. Mice challenged with DNP-BSA showed clear anaphylactic responses. BIS significantly reduced the exudation of Evans blue on visual inspection (Figure 5A) and the Evans blue dye extracted from the reaction site of the ear (Figure 5C). In addition, toluidine blue staining of the ear was performed to observe mast-cell features. After stimulation with DNP-BSA, mast cells (red arrow) migrated to the section of the ear, and many mast cells were activated (Figure 5B), whereas BIS significantly reduced the number of mast cells (Figure 5D) and particularly inhibited mast-cell activation (Figure 5E).

### 2.5. Effects of BIS in IgE/Ag-Stimulated BMMCs

**Figure 6:**
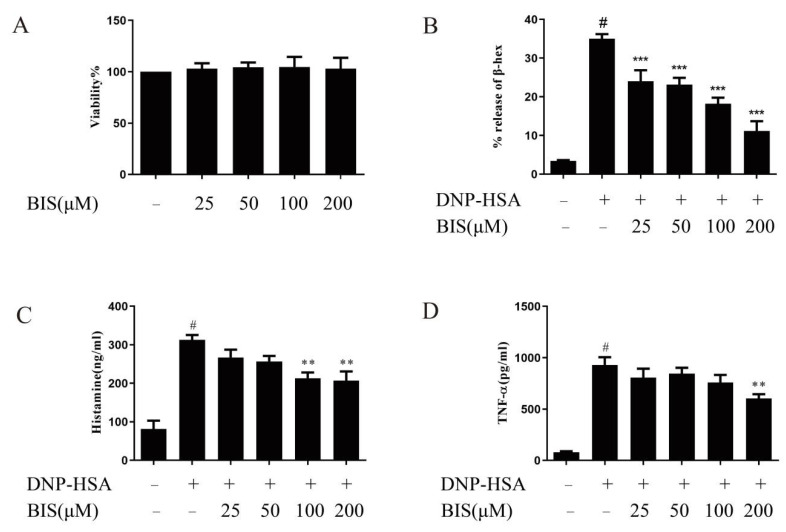
Effect of BIS on IgE/Ag-stimulated BMMCs. (**A**) BMMCs were incubated with different concentrations of BIS and cell viability was determined by XTT assay. IgE-sensitized BMMCs pre-treated with or without BIS for 30 min. Then, the cells were treated with DNP-HSA for stimulation. The release of β-hex (**B**) and histamine (**C**) were measured with DNP-HSA for 30 min. (**D**) Levels of TNF-α were measured with DNP-HSA for 24 h by ELISA. Data represent the mean ± SEM, *n* = 3. ^#^ *p* < 0.05 vs. Control; ** *p* < 0.01, *** *p* < 0.001 vs. Model.

Before investigating the effect of BIS on mast-cell activation, we used an XTT assay to examine the cytotoxicity of BIS on BMMCs. After pre-treatment with BIS at various concentrations (25, 50, 100, and 200 µM) for 24 h, BIS did not exhibit any cytotoxicity, even at 200 µM in the XTT test of BMMCs (Figure 6A). Therefore, we used BIS at concentrations up to 200 µM for all in vitro experiments. To determine the effect of BIS on BMMC degranulation, we first incubated sensitized BMMCs with various concentrations (25, 50, 100, and 200 µM) of BIS for 30 min, and then the cells were stimulated with DNP-HSA for another 30 min. After stimulation with DNP-HSA, BMMCs were activated to release β-hex and histamine. Our results show that BIS treatment significantly inhibited β-hex (Figure 6B) and histamine (Figure 6C) release in a dose-dependent manner. MC-derived TNF was crucial for contact hypersensitivity-induced skin inflammation [29]. Therefore, we measured the effect of BIS on TNF-α release. BIS were pre-treated with IgE-sensitized BMMCs for 30 min, and then stimulated with DNP-HSA for 24 h. We found that BIS significantly reduced TNF-α secretion at 200 µM (Figure 6D). Collectively, these results supported that BIS plays a role in inhibiting mast-cell activation.

### 2.6. Effect of BIS on the MAPK and NF-κB Signaling Pathway in IgE/Ag-Stimulated BMMCs

**Figure 7:**
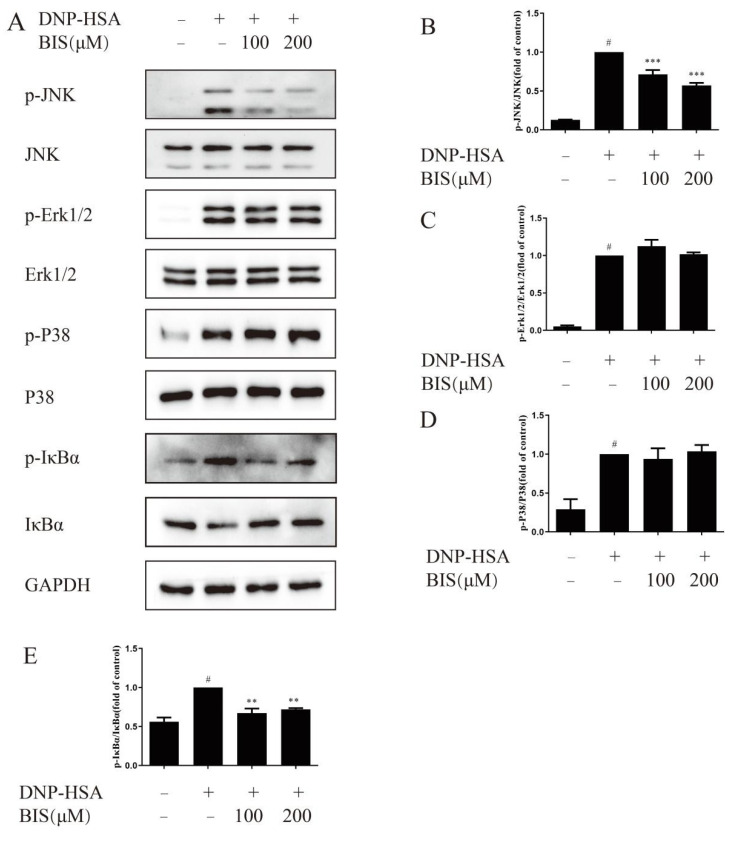
Effect of BIS on MAPK and NF-κB signaling pathways in IgE/Ag-stimulated BMMCs. Cells were sensitized with IgE overnight and incubated with or without BIS for 30 min. Then, cells were stimulated with DNP-HSA for 15 min. Cell protein expression was detected and analyzed by western blotting. (**A**) The expression of phosphorylation of JNK, Erk1/2, P38, and IκBα and JNK, Erk1/2, P38, and IκBα were determined with western blotting. Densitometric analysis of p-JNK/JNK (**B**), p-Erk1/2/Erk1/2 (**C**), p-P38/P38 (**D**), and p-IκBα/IκBα (**E**). Data represent the mean ± SEM, *n* = 3. ^#^ *p* < 0.05 vs. Control; ** *p* < 0.01, *** *p* < 0.001 vs. Model.

MAPK signaling is an important pathway in inflammation response in mast cells. Here, we tested the effect of BIS on p-JNK, p-Erk1/2, and p-P38 by western blotting. As shown in Figure 7A, p-JNK, p-Erk1/2 and, p-P38 all increased after DNP-HSA stimulation for 15 min in BMMCs. BIS dose-dependently attenuated p-JNK (Figure 7A,B); however, p-Erk1/2 (Figure 7A,C) and p-P38 (Figure 7A,D) did not change after treatment with BIS. Most studies on BIS have focused on the NF-κB pathway as a common target in explaining its biological activities [25]. Therefore, we measured the effect of BIS on p-IκBα and total IκBα. IκBα was phosphorylated and decomposed after DNP-HSA stimulation for 15 min. BIS had a suppressive effect on both IκBα phosphorylation (Figure 7A,E) and degradation (Figure 7A,E).

## 3. Discussion

BIS has generated considerable economic interest since it is used as an active ingredient in commercial products, and it has been shown to have multiple biologic activities [18]. In this study, we firstly report that BIS inhibits DNCB-induced AD-like symptom via inhibiting mast-cell degranulation.

AD is one of the most common chronic inflammatory skin diseases, which is stimulated by environmental factors such as mites and exposure to allergens [30,31]. Repeated stimulation by environmental factors leads to severe allergic inflammatory responses, and then leads to erythema, thickened and broken skin, and abnormal immune responses [32,33]. Here, we found that BIS inhibited DNCB-induced skin inflammation, including significant decrease in dermatitis score (Figure 1C), amelioration of the thickness of both the epidermis (Figure 2C) and dermis (Figure 2D), and inhibition of abnormal immune responses such as spleen index (Figure 3A).

IL-4 is an important cytokine in AD [28]. It can drive T-cell development into Th2 cells and induce the release of type-2-related cytokines and chemokines [34]. Subsequently, B cells produce IgE with the stimulation of type-2-related cytokines. Then, IgE binds to high-affinity receptors on the surface of mast cells and activates mast cells, which also release IL-4 and constitute an immediate hypersensitivity reaction in AD. In this study, we found BIS significantly inhibited the skin IL-4 release (Figure 3B) without affecting the release of IgE in either the dorsal skin (Figure 3C) or serum (Figure 3D). Thus, we felt BIS inhibited the production of IL-4 which was released by mast cells but not T cells, and the anti-AD activity of BIS is related to the inhibition of mast-cell activation. As expected, we found that BIS reduced the infiltration and activation of mast cells in the dorsal skin in DNCB-induced BALB/c mice (Figure 4). In addition, we found that BIS suppressed anaphylactic responses in ICR mice caused by mast-cell activation (Figure 5A,C). We confirmed that BIS also inhibited the infiltration and activation of mast cells in the PCA assay (Figure 5B,D,E). Furthermore, we measured the effect of BIS on mast cells in vitro. We found that BIS significantly reduced the release of β-hex, histamine, and TNF-α without cytotoxicity (Figure 6). Thus, we believe that BIS improves dermatitis by stabilizing mast cells.

MAPK signaling is vital for the FcεRI-mediated production of cytokines in mast cells [35,36,37]. In particular, the study showed that the JNK pathway is critical for the release of cytokines, such as IL-4, IL-6, and TNF-α [38]. NF-κB is another distal signaling pathway during mast-cell activation and is a pivotal transcription factor associated with AD [39]. Previous studies have shown that BIS inhibited MAPK and NF-κB pathways to improve outcomes in variety of diseases such as osteoarthritis [25] and lipopolysaccharide-induced pulmonary inflammation [40]. For these reasons, we investigated the involvement of BIS in IgE/Ag-induced NF-κB and MAPK pathways. We found that BIS dose-dependently attenuated the phosphorylation of IκBα and further suppressed the degradation of IκBα (Figure 7A,E). However, BIS did not alter the phosphorylation of p38, MAPK, and Erk1/2 (Figure 7A,C,D) but significantly suppressed activation of JNK (Figure 7A,B).

Studies reported that BIS improved multiple diseases via MAPK and NF-κB pathways [41,42]. BIS can improve nephrotoxicity by mitigating inflammation and oxidative stress through the inhibition of NF-κB activation [43]. BIS-loaded lipid-core nanocapsules suppressed lipopolysaccharide-induced acute respiratory distress syndrome by inhibition of the MAPK pathway [40]. Furthermore, BIS alleviated isoproterenol (ISO)-induced myocardial infarction (MI) through NF-κB/MAPK signaling pathways [19]. Therefore, MAPK and NF-κB may be the target of BIS in different diseases. Here, we focus on the effect of BIS on JNK and NF-κB signaling. The study shows that the molecular docking indicated that BIS may have a high affinity for JNK at −6.3 kcal/mol. BIS functions by occupying the inhibitory binding pockets of JNK by interacting with GLU-109 of JNK, and it was completely encapsulated in the inhibitory region of corresponding proteins [25]. In addition, this study also showed that BIS may have a high affinity for p65 at −3.3 kcal/mol which is moderately higher. Thus, we speculate that BIS may directly bind to JNK while affecting the activation of NF-κB through other targets.

Proliferator-activated receptor gamma (PPAR-γ) plays an anti-inflammatory role in allergies and is expressed in mast cells [44]. PPAR-γ exerts an anti-inflammatory effect by inhibiting NF-κB activation. For example, PPAR-γ agonists improved inflammatory bowel disease symptoms by inhibiting NF-κB activation [45]. PPAR-γ agonists reduced the expression of TNF-α and IL-4 through down-regulation of NF-κB activation in mast-cell-related allergic diseases [46,47]. Therefore, PPAR-γ/NF-κB signaling may be important in mast-cell activation. BIS mitigated colonic inflammation by stimulating PPAR-γ transcription factor expression and inhibiting NF-κB signaling [25]. In addition, this study reported that BIS may have a strong binding affinity (−7.4 kcal/mol) for the PPAR-γ binding site. Thus, we think BIS may affect the NF-κB signaling in mast cells by promoting PPAR-γ activation.

**Figure 8:**
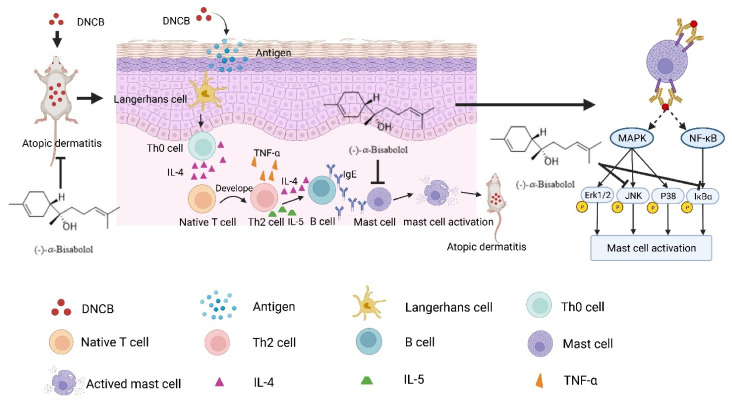
BIS-attenuated degranulation of mast cell by inhibiting MAPK and NF-κB pathway in DNCB-induced atopic dermatitis.

Collectively, we propose a model to explain the anti-AD effects of BIS (Figure 8). In summary, we first demonstrated the anti-AD effect of BIS by inhibiting JNK and NF-κB pathways in mast cells. Our results suggest BIS might serve as a promising therapeutic agent particularly for chronic AD patients, since it has low toxicity as classified by the United States Food and Drug Administration. Nevertheless, to further confirm the effectiveness of BIS in AD, studies with larger sample sizes and research on human samples should be carried out.

## 4. Materials and Methods

### 4.1. Materials

BIS, Mouse anti-dinitrophenyl (DNP) IgE, 2,4-dinitrochlorobenzene (DNCB), glycine, cell proliferation Kit II, 4-Nitrophenyl N-acetyl-β-D-glucosaminide (p-NAG), bovine serum albumin (BSA), ketotifen, formamide, Evans Blue, and o-phthaldialdehyde were purchased from Sigma-Aldrich (ST. Louis, MO, USA). The 2,4-dinitrophenyl-human serum albumin (DNP-HSA) and 2,4-dinitrophenyl-bovine serum albumin (DNP-BSA) were from Biosearch technology. The antibodies specific for p-JNK (Thr183/Tyr185), JNK, p-Erk1/2 (Thr202/Tyr204), Erk1/2, p-P38 (Thr180/Tyr182), P38, p-IκBα (Ser32), IκBα, and normal rabbit IgG were from Cell Signaling Technology (Danvers, MA, USA). The antibodies specific for GAPDH were from Proteintech (Chicago, USA). RIPA and BCA protein assay kits were from Beyotime (Beijing, China). Fetal Bovine Serum (FBS), mouse TNF alpha ELISA kit, mouse IL-4 ELISA kit, and mouse IgE ELISA kit were from Thermo Fisher. Recombinant mouse IL-3 and recombinant mouse SCF/c-kit ligand were from RD systems China. ECL Immobilon Western Chemiluminescent HRP Substrate was from Millipore (Billerica, USA). Compound dexamethasone acetate cream was purchased from CR SAN JIU (Shenzhen, China).

### 4.2. Animals

Mice were purchased from the B&K Laboratory Animal Corp. Ltd. (Shanghai, China) and housed in a specific pathogen-free (SPF) animal room. The animals were housed at the Animal Laboratory Building of Shanghai University of Traditional Chinese Medicine under a constant temperature (20–26 °C) and 40%–60% humidity with a 12 h light–dark cycle. All animal experiments were approved by the experimental Animal Ethics Committee of Shanghai University of Traditional Chinese Medicine (PZSHUTCM210528004, PZSHUTCM201016009).

### 4.3. DNCB-Induced Atopic Dermatitis in BALB/c Mice

Female BALB/c mice were randomly divided into five groups as follows: control group was sensitized and challenged with acetone:olive oil in a 3:1 ratio; model group was sensitized and challenged with DNCB in a 3:1 ratio of acetone:olive oil; BIS groups was challenged with DNCB and treated topically with 200 mg/kg or 400 mg/kg BIS; and dexamethasone (DEX) group also was challenged with DNCB and treated topically with dexamethasone cream which contains dexamethasone 1.125 mg/kg to cover the dorsal skin. The dorsal skin was shaved with an electric clipper and hair removal creams. To sensitize the skin, 200 µL of 1% DNCB in acetone:olive oil (3:1) was applied to the shaved area twice a week on the first week of the experiment. Then, for the challenge process, 200 µL of 0.4% DNCB was applied to the dorsal skin twice a week on experimental weeks 2–6. BIS dissolved in 3% Tween 80 presents as liquid. Using a micropipette to smear 200 µL BIS to the 5 cm^2^ dorsal skin of mice, BIS and DEX treatments were applied to the dorsal skin of the mice five times a week on experimental weeks 3–6. Mice were sacrificed on the 42nd day. Spleens were weighted to calculate the spleen index (spleen weight/body weight). Blood serum and the dorsal skin were collected. The skin thickness of mice was measured with a Vernier caliper. Then, skin tissues were fixed with 4% paraformaldehyde and embedded in paraffin. Sections were stained with hematoxylin and eosin (H&E) as well as toluidine blue. The experimental design is shown in Figure 1A.

### 4.4. Assessment of the Severity of AD-like Skin Lesions

The degree of atopic dermatitis in AD-like lesions was detected on experimental weeks 3–6. Four major classes of symptoms in AD were identified as erythema or hemorrhage, edema, excoriation or erosion, and scarring or dryness [48]. The score was denoted as 0, 1, 2, or 3 according to the severity of each symptom (none, mild, moderate, or severe). The individual score was the sum of scores from each symptom.

### 4.5. Passive Cutaneous Anaphylaxis

PCA was performed as described in a previous study [49]. In brief, male ICR mice (18–20 g) received an oral administration of 200 mg/kg, 400 mg/kg BIS, or 10 mg/kg ketotifen on the experimental days 1–3. One (1) µg of anti-mouse-IgE was intradermally injected into the ear of mice on the experimental day 2. After 24 h, the mice were challenged for 30 min by intravenous injection of 100 µg DNP-BSA in 300 µL saline. Then, ear tissues were fixed in 4% paraformaldehyde and embedded in paraffin. Tissue sections were stained with toluidine blue. Alternatively, the mice were challenged for 30 min by intravenous injection of 100 µg DNP-BSA in 300 µL saline containing 0.5% Evans blue. Evans blue was extracted with 300 µL of formamide at room temperature for 24 h and measured by absorbance at 630 nm.

### 4.6. Bone-Marrow-Derived Mast Cells (BMMCs) Culture

Bone-marrow cells were isolated from BALB/c mice. The bone-marrow cells were rinsed with chilled PBS and centrifuged at 200 × *g* for 5 min. Then, cells were resuspended in RPMI 1640 medium supplemented with 10% FBS, 100 U/mL penicillin/streptomycin, 1 mM sodium pyruvate, 0.1 mM nonessential amino acids, 50 µM β-mercaptoethanol, and 10 ng/mL recombinant mouse IL-3 and SCF. Cells were fed every 7 days. After 4 weeks, cell purity was determined by measuring CD117 and FcεRIα expression through flow cytometry: 95% of cells were positive for both CD117 and FcεRIα.

### 4.7. Cell Viability Analysis

Cell viability was measured with a cell proliferation Kit II according to the manufacturer’s protocol. Cells were seeded in 96-well plates at a density of 5 × 10^5^ cells per well and then incubated with different concentrations of BIS (25, 50, 100, and 200 µM) for 24 h. Then, 50 µL of (2,3-bis (2-methoxy-4-nitro-5-sulfophenyl)−5-[(phenylamino)carbonyl]-2H-tetrazolium hydroxide) XTT solution was added to each well and incubated for 4 h at 37 °C with 5% CO_2_. The absorbance was measured at 492 nm and 690 nm by a microplate reader.

### 4.8. β-Hexosaminidase Release Assay

BMMCs at concentration of 1 × 10^6^ cells/mL were sensitized overnight with 0.5 µg/mL of IgE. The BMMCs were resuspended in HEPES buffer containing 0.4% BSA. Then, cells were treated with different concentrations of BIS (25, 50, 100, and 200 µM) and then incubated for 30 min at 37 °C. Finally, the cells were stimulated with 50 ng/mL of DNP-HSA for 30 min. Then, mast-cell degranulation was assessed by measuring β-hex release. First, 50 µL of supernatant and pellet were removed to another plate. Then, 50 µL of 4-nitrophenyl N-acetyl-β-D-glucosaminide (p-NAG) was added for 90 min at 37 °C. Finally, glycine (pH 10.7) was added to stop the reaction. The absorbance was measured at 405 and 570 nm. The calculation formula was: % release of β-hex = 100 × supernatant absorbance/(0.5 × supernatant absorbance + pellet absorbance)

### 4.9. Histamine Assay

BMMCs at concentration of 2 × 10^6^ cells/mL were sensitized overnight with 0.5 µg/mL of anti-DNP-IgE. Then, the cells were resuspended in HEPES buffer and treated with different concentrations of BIS (25, 50, 100, and 200 µM) for 30 min at 37 °C and 5% CO_2_. Finally, cells were treated with 0.1 µg/mL of DNP-HSA for 30 min. After stimulation, cells were centrifuged at 200 × *g* for 5 min to obtain the supernatant. The histamine standard curve (7.8–500 ng/mL) was freshly prepared. Sixty (60) µL of different concentrations of histamine standards and cell supernatant was transferred to 96-well plate. Twelve (12) µL of 1 M NaOH and 2 µL of 10 mg/mL o-phthalaldehyde were added and reacted for 4 min at room temperature. Then, 6 µL of 3 M HCl was added to terminate the reaction. The fluorescence was detected at A_360_ as the excitation filter and A_450_ as the emission filter.

### 4.10. Enzyme Linked Immunosorbent Assay (ELISA)

BMMCs at concentration of 5 × 10^5^ cells/mL were sensitized overnight with 0.5 µg/mL of IgE and treated with different concentrations of BIS (25, 50, 100, and 200 µM) for 30 min. Then, cells were stimulated with 50 ng/mL of DNP-HSA. After 24 h, the supernatant was collected for ELISA.

The cell supernatant was analyzed for TNF-α; the serum samples were analyzed for IgE. The protein content from skin samples was measured and adjusted to 5 mg/mL for all samples which were analyzed for IL-4 and IgE with an ELISA, according to the manufacturer’s instructions.

### 4.11. Western Blotting

Cells were sensitized with 0.5 µg/mL anti-DNP-IgE overnight and resuspended in HEPES buffer. After incubating with or without BIS at 37 °C for 30 min, the cells were subsequently stimulated with 50 ng/mL DNP-HSA for 15 min. Cells were lysed using protein lysis solution (radioimmunoprecipitation assay (RIPA), protease inhibitor, phosphatase inhibitor A and B, and PMSF). Cell lysates were centrifuged at 12,000 rpm for 15 min. After measuring the protein concentration by using the BCA assay, the samples were adjusted to the same concentration with PBS and 5 × loading buffer. The samples were boiled at 95 °C for 10 min and stored at −80 °C.

Polyacrylamide gel with the appropriate concentration was prepared according to the molecular weight of the protein. The proteins were separated via SDS-PAGE and transferred to PVDF membranes. The membrane was blocked with 5% non-fat milk for 1 h and incubated with a suitable primary antibody at 4 °C overnight. The membrane was washed 5 times with TBST and incubated with anti-rabbit IgG antibody conjugated to horseradish peroxidase (HRP) for 1 h at room temperature. Finally, the membrane was detected using chemiluminescence (ECL) reagents according to the manufacturer’s protocol.

### 4.12. Statistical Analysis

Data were expressed as the means ± standard error of the mean (SEM). Results were analyzed using one-way ANOVA through GraphPad Prism 8.4.3. *p* ≤ 0.05, 0.01, and 0.001 were considered to indicate a statistical significance.

## Data Availability

The data presented in this study are available in this article.

## References

[1] Hou D.D., Di Z.H., Qi R.Q., Wang H.X., Zheng S., Hong Y.X., Guo H., Chen H.D., Gao X.H. (2017). Sea Buckthorn (*Hippophae rhamnoides* L.) Oil Improves Atopic Dermatitis-Like Skin Lesions via Inhibition of NF-kappaB and STAT1 Activation. Ski. Pharm. Physiol..

[2] Coutanceau C., Stalder J.F. (2014). Analysis of correlations between patient-oriented SCORAD (PO-SCORAD) and other assessment scores of atopic dermatitis severity and quality of life. Dermatology.

[3] Weidinger S., Beck L.A., Bieber T., Kabashima K., Irvine A.D. (2018). Atopic dermatitis. Nat. Rev. Dis. Primers.

[4] Sacotte R., Silverberg J.I. (2018). Epidemiology of adult atopic dermatitis. Clin. Derm..

[5] Jiang X., Lan Y., Wei B., Dai C., Gu Y., Ma J., Liu X., Umezawa K., Zhang Y. (2017). External application of NF-kappaB inhibitor DHMEQ suppresses development of atopic dermatitis-like lesions induced with DNCB/OX in BALB/c mice. Immunopharmacol. Immunotoxicol..

[6] Kim G.D., Park Y.S., Ahn H.J., Cho J.J., Park C.S. (2015). Aspartame Attenuates 2, 4-Dinitrofluorobenzene-Induced Atopic Dermatitis-Like Clinical Symptoms in NC/Nga Mice. J. Investig. Derm..

[7] Peng W., Novak N. (2015). Pathogenesis of atopic dermatitis. Clin. Exp. Allergy.

[8] Brunner P.M., Guttman-Yassky E., Leung D.Y. (2017). The immunology of atopic dermatitis and its reversibility with broad-spectrum and targeted therapies. J. Allergy Clin. Immunol..

[9] Brunner P.M., Silverberg J.I., Guttman-Yassky E., Paller A.S., Kabashima K., Amagai M., Luger T.A., Deleuran M., Werfel T., Eyerich K. (2017). Increasing Comorbidities Suggest that Atopic Dermatitis Is a Systemic Disorder. J. Investig. Derm..

[10] Nishi K., Kanayama Y., Kim I.H., Nakata A., Nishiwaki H., Sugahara T. (2019). Docosahexaenoyl ethanolamide mitigates IgE-mediated allergic reactions by inhibiting mast cell degranulation and regulating allergy-related immune cells. Sci. Rep..

[11] Hellman L.T., Akula S., Thorpe M., Fu Z. (2017). Tracing the Origins of IgE, Mast Cells, and Allergies by Studies of Wild Animals. Front. Immunol..

[12] Yosipovitch G., Papoiu A.D. (2008). What causes itch in atopic dermatitis?. Curr. Allergy Asthma Rep..

[13] Holgate S.T. (2000). The role of mast cells and basophils in inflammation. Clin. Exp. Allergy.

[14] Brown J.M., Wilson T.M., Metcalfe D.D. (2008). The mast cell and allergic diseases: Role in pathogenesis and implications for therapy. Clin. Exp. Allergy.

[15] Honda T., Kabashima K. (2019). Prostanoids and leukotrienes in the pathophysiology of atopic dermatitis and psoriasis. Int. Immunol..

[16] Oetjen L.K., Mack M.R., Feng J., Whelan T.M., Niu H., Guo C.J., Chen S., Trier A.M., Xu A.Z., Tripathi S.V. (2017). Sensory Neurons Co-opt Classical Immune Signaling Pathways to Mediate Chronic Itch. Cell.

[17] Gardiner P.J.P. (2007). Complementary, holistic, and integrative medicine: Chamomile. Pediatr. Rev..

[18] Ortiz-Bautista R., García-González L., Ocádiz-González M., Flores-Tochihuitl J., García-Villaseñor A., González-Hernández M., Muñoz-Hernández L., Ortiz-Figueroa M., Ramírez-Anaya M., Reyna-Téllez S. (2017). Matricaria chamomilla (aqueous extract) improves atopic dermatitis-like lesions in a murine model. Rev. Médica Del Inst. Mex. Del Seguro Soc..

[19] Nagoor Meeran M.F., Azimullah S., Laham F., Tariq S., Goyal S.N., Adeghate E., Ojha S. (2020). α-Bisabolol protects against β-adrenergic agonist-induced myocardial infarction in rats by attenuating inflammation, lysosomal dysfunction, NLRP3 inflammasome activation and modulating autophagic flux. Food Funct..

[20] Ortiz M.I., Cariño-Cortés R., Ponce-Monter H.A., Castañeda-Hernández G., Chávez-Piña A.E. (2018). Pharmacological interaction of α-bisabolol and diclofenac on nociception, inflammation, and gastric integrity in rats. Drug Dev. Res..

[21] Barreto R.S.S., Quintans J.S.S., Amarante R.K.L., Nascimento T.S., Amarante R.S., Barreto A.S., Pereira E.W.M., Duarte M.C., Coutinho H.D.M., Menezes I.R.A. (2016). Evidence for the involvement of TNF-alpha and IL-1beta in the antinociceptive and anti-inflammatory activity of Stachys lavandulifolia Vahl. (Lamiaceae) essential oil and (-)-alpha-bisabolol, its main compound, in mice. J. Ethnopharmacol..

[22] Cavalcante H.A.O., Silva-Filho S.E., Wiirzler L.A.M., Cardia G.F.E., Uchida N.S., Silva-Comar F.M.S., Bersani-Amado C.A., Cuman R.K.N. (2020). Effect of (-)-alpha-Bisabolol on the Inflammatory Response in Systemic Infection Experimental Model in C57BL/6 Mice. Inflammation.

[23] Javed H., Meeran M., Azimullah S., Bader Eddin L., Dwivedi V., Jha N., Ojha S.J.B. (2020). α-Bisabolol, a Dietary Bioactive Phytochemical Attenuates Dopaminergic Neurodegeneration through Modulation of Oxidative Stress, Neuroinflammation and Apoptosis in Rotenone-Induced Rat Model of Parkinson’s disease. Biomolecules.

[24] Murata Y., Kokuryo T., Yokoyama Y., Yamaguchi J., Miwa T., Shibuya M., Yamamoto Y., Nagino M. (2017). The Anticancer Effects of Novel alpha-Bisabolol Derivatives Against Pancreatic Cancer. Anticancer Res..

[25] Xu C., Sheng S., Dou H., Chen J., Zhou K., Lin Y., Yang H. (2020). α-Bisabolol suppresses the inflammatory response and ECM catabolism in advanced glycation end products-treated chondrocytes and attenuates murine osteoarthritis. Int. Immunopharmacol..

[26] Veraldi S., De Micheli P., Schianchi R., Lunardon L. (2009). Treatment of pruritus in mild-to-moderate atopic dermatitis with a topical non-steroidal agent. J. Drugs Dermatol..

[27] Wang Y., Cao S., Yu K., Yang F., Yu X., Zhai Y., Wu C., Xu Y. (2019). Integrating tacrolimus into eutectic oil-based microemulsion for atopic dermatitis: Simultaneously enhancing percutaneous delivery and treatment efficacy with relieving side effects. Int. J. Nanomed..

[28] Homey B., Steinhoff M., Ruzicka T., Leung D.Y. (2006). Cytokines and chemokines orchestrate atopic skin inflammation. J. Allergy Clin. Immunol..

[29] Dudeck J., Kotrba J., Immler R., Hoffmann A., Voss M., Alexaki V.I., Morton L., Jahn S.R., Katsoulis-Dimitriou K., Winzer S. (2021). Directional mast cell degranulation of tumor necrosis factor into blood vessels primes neutrophil extravasation. Immunity.

[30] Choi C.Y., Kim Y.H., Oh S., Lee H.J., Kim J.H., Park S.H., Kim H.J., Lee S.J., Chun T. (2017). Anti-inflammatory potential of a heat-killed Lactobacillus strain isolated from Kimchi on house dust mite-induced atopic dermatitis in NC/Nga mice. J. Appl. Microbiol..

[31] Trikamjee T., Basera W., Botha M., Facey-Thomas H., Gaunt B., Genuneit J., Gray C., Hadebe S., Hlela C., Kirstein F. (2021). Associations between Environmental dust composition and Atopic Dermatitis in urban and rural settings. Pediatric Allergy Immunol..

[32] Kim T.Y., Park N.J., Jegal J., Choi S., Lee S.W., Hang J., Kim S.N., Yang M.H. (2019). Chamaejasmine Isolated from Wikstroemia dolichantha Diels Suppresses 2,4-Dinitrofluoro-benzene-Induced Atopic Dermatitis in SKH-1 Hairless Mice. Biomolecules.

[33] Lee S.Y., Park N.J., Jegal J., Jo B.G., Choi S., Lee S.W., Uddin M.S., Kim S.N., Yang M.H. (2020). Suppression of DNCB-Induced Atopic Skin Lesions in Mice by Wikstroemia indica Extract. Nutrients.

[34] Xu M., Dong C. (2017). IL-25 in allergic inflammation. Immunol. Rev..

[35] Chen J.J., Zhang L.N., Wang H.N., Xie C.C., Li W.Y., Gao P., Hu W.Z., Zhao Z.F., Ji K. (2021). FAK inhibitor PF-431396 suppresses IgE-mediated mast cell activation and allergic inflammation in mice. Biochem. Pharmacol..

[36] Niu L., Wei J., Li X., Jin Y., Shi X. (2021). Inhibitory activity of narirutin on RBL-2H3 cells degranulation. Immunopharmacol. Immunotoxicol..

[37] Yousef M., Crozier R.W.E., Hicks N.J., Watson C.J.F., Boyd T., Tsiani E., MacNeil A.J. (2020). Attenuation of allergen-mediated mast cell activation by rosemary extract (*Rosmarinus officinalis* L.). J. Leukoc. Biol..

[38] Garrington T.P., Ishizuka T., Papst P.J., Chayama K., Webb S., Yujiri T., Sun W., Sather S., Russell D.M., Gibson S.B. (2000). MEKK2 gene disruption causes loss of cytokine production in response to IgE and c-Kit ligand stimulation of ES cell-derived mast cells. EMBO J..

[39] Kang Y.M., Kim H.M., Lee M., An H.J. (2021). Oleanolic Acid Alleviates Atopic Dermatitis-like Responses In Vivo and In Vitro. Int. J. Mol. Sci..

[40] D’Almeida A.P.L., Pacheco de Oliveira M.T., de Souza É.T., de Sá Coutinho D., Ciambarella B.T., Gomes C.R., Terroso T., Guterres S.S., Pohlmann A.R., Silva P.M. (2017). α-bisabolol-loaded lipid-core nanocapsules reduce lipopolysaccharide-induced pulmonary inflammation in mice. Int. J. Nanomed..

[41] Kim S., Jung E., Kim J.H., Park Y.H., Lee J., Park D. (2011). Inhibitory effects of (-)-α-bisabolol on LPS-induced inflammatory response in RAW264.7 macrophages. Food Chem. Toxicol..

[42] Chen W., Hou J., Yin Y., Jang J., Zheng Z., Fan H., Zou G.J.B. (2010). Alpha-Bisabolol induces dose- and time-dependent apoptosis in HepG2 cells via a Fas- and mitochondrial-related pathway, involves p53 and NFkappaB. Biochem. Pharm..

[43] Zaaba N., Beegam S., Elzaki O., Yasin J., Nemmar B., Ali B., Adeghate E., Nemmar A.J.B. (2022). The Nephroprotective Effects of α-Bisabolol in Cisplatin-Induced Acute Kidney Injury in Mice. Biomedicines.

[44] Sugiyama H., Nonaka T., Kishimoto T., Komoriya K., Tsuji K., Nakahata T.J.F. (2000). Peroxisome proliferator-activated receptors are expressed in mouse bone marrow-derived mast cells. FEBS Lett..

[45] Ramakers J.D., Verstege M.I., Thuijls G., Te Velde A.A., Mensink R.P., Plat J. (2007). The PPARgamma agonist rosiglitazone impairs colonic inflammation in mice with experimental colitis. J. Clin. Immunol..

[46] Yao L., Gu Y., Jiang T., Che H. (2022). Inhibition effect of PPAR-γ signaling on mast cell-mediated allergic inflammation through down-regulation of PAK1/NF-κB activation. Int. Immunopharmacol..

[47] Gu Y., Guo X., Sun S., Che H. (2022). High-Fat Diet-Induced Obesity Aggravates Food Allergy by Intestinal Barrier Destruction and Inflammation. Int. Arch. Allergy Immunol..

[48] Matsuda H., Watanabe N., Geba G.P., Sperl J., Tsudzuki M., Hiroi J., Matsumoto M., Ushio H., Saito S., Askenase P.W. (1997). Development of atopic dermatitis-like skin lesion with IgE hyperproduction in NC/Nga mice. Int. Immunol..

[49] Qian F., Zhang L., Lu S., Mao G., Guo F., Liu P., Xu J., Li Y. (2019). Scrodentoid A Inhibits Mast Cell-Mediated Allergic Response by Blocking the Lyn-FcεRIβ Interaction. Front. Immunol..

